# Electrocortical Activity in Older Adults Is More Influenced by Cognitive Task Complexity Than Concurrent Walking

**DOI:** 10.3389/fnagi.2021.718648

**Published:** 2022-01-24

**Authors:** Carolina Vila-Chã, Cláudia Vaz, Anderson Souza Oliveira

**Affiliations:** ^1^Polytechnic of Guarda, Guarda, Portugal; ^2^Research Center in Sports Sciences, Health Sciences and Human Development, CIDESD, Vila Real, Portugal; ^3^Department of Materials and Production, Aalborg University, Aalborg, Denmark

**Keywords:** dual-task, cognitive load, physical activity, decision-making, walking, electroencephalography

## Abstract

Human cognitive-motor performance largely depends on how brain resources are allocated during simultaneous tasks. Nonetheless, little is known regarding the age-related changes in electrocortical activity when dual-task during walking presents higher complexity levels. Thus, the aim of this study was to investigate whether there are distinct changes in walking performance and electrocortical activation between young and older adults performing simple and complex upper limb response time tasks. Physically active young (23 ± 3 years, *n* = 21) and older adults (69 ± 5 years, *n* = 19) were asked to respond as fast as possible to a single stimuli or a double stimuli appearing on a touch screen during standing and walking. Response time, step frequency, step frequency variability and electroencephalographic (EEG) N200 and P300 amplitudes and latencies from frontal central and parietal brain regions were recorded. The results demonstrated that older adults were 23% slower to respond to double stimuli, whereas younger adults were only 12% slower (*p* < 0.01). The longer response time for older adults was accompanied by greater step frequency variability following double-stimuli presentations (*p* < 0.01). Older adults presented reduced N200 and P300 amplitudes compared to younger participants across all conditions (*p* < 0.001), with no effects of posture (standing vs walking) on both groups (*p* > 0.05). More importantly, the P300 amplitude was significantly reduced for older adults when responding to double stimuli regardless of standing or walking tasks (*p* < 0.05), with no changes in younger participants. Therefore, physically active older adults can attenuate potential walking deficits experienced during dual-task walking in simple cognitive tasks. However, cognitive tasks involving decision making influence electrocortical activation due to reduced cognitive resources to cope with the task demands.

## Introduction

The combination of cognitive and motor tasks reduces the performance of at least one of the two tasks regardless of age, as it is related to the allocation of resources during simultaneous tasks (Malcolm et al., 2015; Vasquez et al., 2016). An increase in task complexity might rise cognitive-motor effort and reduce attentional reserve (Al-Yahya et al., 2011; De Sanctis et al., 2014; Gentili et al., 2018). Such conditions can lead to reduced cognitive processing efficiency, ultimately reducing motor performance and the ability to react to unexpected events (Bayot et al., 2018; Gentili et al., 2018). This may be particularly problematic for older adults during daily life situations that require walk and concurrent demanding cognitive tasks (Lindenberger et al., 2000). Despite physically active older adults can perform normal walking and simple precision stepping similarly to young adults, they became slower than young adults when the complexity of the precision stepping task increased (Oliveira et al., 2018b). Such results suggest that even older adults that have an active physical lifestyle—an important factor to attenuate natural age-related decay in motor and cognitive performance—experience a greater decline in the performance of complex dual-tasks than young adults (Plummer et al., 2015). Therefore, cognitive task complexity may be the greatest factor in reducing motor performance during dual-tasking in older adults. The compromise in performance caused by cognitive-motor interference is related to limited neuronal resources to cope with the cognitive demands (Malcolm et al., 2015; Vasquez et al., 2016). However, little is known regarding the age-related changes in electrocortical activity when dual-task during walking presents different complexity levels.

It has been shown that aging reduces cortical gray matter volumes in the frontal and temporal regions, which has been associated with impairments in memory, divided attention, and decision-making (Dickstein et al., 2007; Kennedy and Raz, 2009). Moreover, the performance of concurrent cognitive and motor tasks is degraded in people presenting reduced cortical gray matter volume (Malcolm et al., 2015; Maidan et al., 2019). The implications of such alterations in the cognitive-motor interference during walking have been exemplified by Beurskens et al. (2014), who reported decreased prefrontal cortex activation associated with dual-task load during walking in older adults, but not in young adults. These findings suggested a shift of the processing resources from the prefrontal cortex to other brain regions due to the challenge of walking while performing a visually demanding task (Beurskens et al., 2014). Nonetheless, most studies used near infra-red spectroscopy (fNIRS), restricting the results to the frontal brain region at a low sampling frequency. Therefore, it is highly relevant to access multiple brain regions simultaneously using high temporal resolution to further explore how the brain modulated concomitant cognitive and motor tasks during walking.

Surface electroencephalography (EEG) allows assessing changes in electrocortical activity in multiple brain regions within short periods of time, even during walking (Malcolm et al., 2015; Oliveira et al., 2018a). Malcolm et al. (2015) reported that older adults present reduced accuracy when performing the Go/No-Go task during walking when compared to standing. The reduced accuracy in older adults was accompanied by delayed and reduced P300 amplitude during walking. In contrast, young adults maintained their accuracy during walking, whereas demonstrating changes in electrocortical activity at early (e.g., reduced N200 amplitude) and later (e.g., earlier P300 latency) stages of task completion during walking. Conversely, Maidan et al. (2019) reported similar P300 amplitude in the prefrontal area and increased P300 latency for both young and old adults when performing an oddball discrimination task while walking. Still, older adults presented longer P300 latencies than young adults. Despite the relevance of using stimulus/response paradigms to investigate age-related changes in brain activity, different paradigms influence the comparison across EEG experiments, as they may involve different cortical circuits. Interestingly, regular physical activity is advantageous for aging, but studies on neural activation during dual-task paradigms did not control this critical factor between younger and older adults (Malcolm et al., 2015; Maidan et al., 2019). Physically active older adults may have neural advantages in performing the motor component of combined cognitive-motor tasks, being necessary to set physical activity as inclusion criteria if compared to young and physically active individuals (Barnes, 2015; Pollock et al., 2015; Oliveira et al., 2018b). Therefore, it is relevant to determine age-related changes in electrocortical activity and walking performance deficits under increasing complexity levels imposed by decision-making tasks in physically active individuals.

This study aimed to investigate whether there are distinct changes in walking performance and electrocortical activity between physically active young and older adults performing cognitively simple and complex upper limb response time task. It was hypothesized that increases in the cognitive complexity of the upper limb response time task during walking would have greater impact on the response time, walking stability, and electrocortical activity of physically active older adults when compared to young adults.

## Materials and Methods

### Participants

Forty clinically healthy participants, 21 younger adults (age: 23 ± 3 years, height: 169 ± 9 cm, mass: 68 ± 9 kg) and 19 older adults (age: 69 ± 5 years, height: 165 ± 9 cm, mass: 71 ± 10 kg) participated in this study. Young adults practiced team sports (handball, football, volleyball, and basketball), running, and resistance training 4–5 times/week, totaling 4–6 training hours/week. Older adults were enrolled in the Guarda + 65 physical activity program, practicing water fitness activities, group classes (aerobic exercises, stretching), and resistance training 3–5 times/week, totaling 3–5 training hours/week. Exclusion criteria for this study were: visual and walking impairments, vestibular dysfunctions, a history of lower back and/or lower-extremities pain and/or injuries in the past 6 months. In addition, participants should not have engaged in any type of cognitive training activities such as Lumosity, Fit Brains Trainer, Cognito, and others for the past 6 months. Written informed consent was obtained from all the participants prior to testing. The study was approved by the Polytechnic of Guarda Committee on Research Ethics (N.°1/2019), and all methods conformed to the standards of the Declaration of Helsinki.

### Experimental Protocol

In a single session, participants performed a response time task as described elsewhere (Oliveira et al., 2018b). The task consisted of reacting to the appearance of stimulus presented on the touch screen by tapping in specific colored rectangles. The stimulus presentation and response time recording were conducted through a custom MATLAB^®^ script (R2016b, Mathworks Inc., Natick, MA United States). Both groups performed the task during standing (STAND) and while walking on a treadmill at the preferred speed (WALK). Moreover, there were two types of stimuli: a single stimulus (SINGLE) in which participants should react to a single 6 × 8 cm rectangle consistently appearing in the center of the screen; and (2) double stimuli (DOUBLE), in which participants should react to the appearance of two 6 × 8 cm rectangles of different colors appearing side-by-side in the center of the screen. In the DOUBLE condition, two pairs of colors were displayed: green and yellow as well as green and red. There was only one correct color for each combination: if green and yellow clues appeared, participants should touch the yellow rectangle; if green and red clues appeared, participants should touch the green rectangle. For each stimulus presentation, a fixation cross was presented in the center of the screen, indicating that the participant should focus and be ready to react. The fixation cross was replaced by the stimulus after a random period between 2 and 5 s, and participants were instructed to touch the target stimulus as fast as possible. If the participant touched the wrong rectangle, the attempt was marked as a mistake. This experimental paradigm was chosen as it can reliably assess the readiness to respond to a sensory stimulus using the upper limbs, while it was also possible to assess this motor readiness during treadmill walking. Therefore, it was possible to isolate effects of task complexity (SINGLE vs. DOUBLE) and posture (STAND vs. WALK).

Participants were allowed to familiarize to SINGLE and DOUBLE conditions prior to the data collection. For each condition, four sets of 16 responses were recorded (64 trials in total). All trials were visually inspected to assure that the response times were not related to mistaken trials. The trials marked as mistakes were excluded from the analysis. The order of the tasks (STAND vs. WALK) and the order of the conditions (SINGLE vs. DOUBLE) were randomized for each subject. In addition, the positioning of the color order of the rectangles in the double stimulus condition was randomized.

### Electroencephalographic and Head Acceleration Recordings

Electroencephalography data were recorded using a wireless 32-channel EEG system (LiveAmp, Brain Products Inc., Gilching, Germany). In order to minimize the influence of head motion on the EEG recordings, we used an EEG system with active electrodes, fixated the EEG cables with straps to minimize their motion, and also placed a stretchable mesh cap that assisted in maintaining the electrodes with the decided contact to the scalp across the recording. The channels recorded in this experiment were: FP1, FP2, Fz, C3, C4, Cz, and Pz. The sampling rate was set to 500 Hz. This EEG system is a gel-based system with active electrodes. Head accelerations were acquired in the vertical, anterior-posterior and medial-lateral directions simultaneously with the EEG recordings sampled at 500 Hz. The accelerometer was in-built in the EEG system and was fixed at the head embedded in the EEG cap at the basis of the cranium.

### Data Analysis—Head Acceleration

The accelerations data (in *g* force) were low-pass filtered with a cutoff frequency of 60 Hz using a second order Butterworth filter (Hennig and Lafortune, 1991). The negative peak vertical accelerations in the vertical direction were identified across the continuous recordings. Each of these negative peak accelerations would represent the peak acceleration applied to the head during each stance period of walking (Kline et al., 2015; Snyder et al., 2015). Therefore, these negative peak accelerations can be used to describe the number of steps performed during walking. The six steps preceding a stimulus presentation during walking were defined as walking before stimulus (PRE), whereas the 4 steps immediately after a stimulus presentation were defined as after stimulus (POST). For each participant, the step frequency (steps/minute) and the coefficient of variation of the step frequency (SF_*CV*_ in %) were computed for PRE and POST conditions, for both single and double stimuli conditions.

### Data Analysis—Electroencephalographic

**FIGURE 1:**
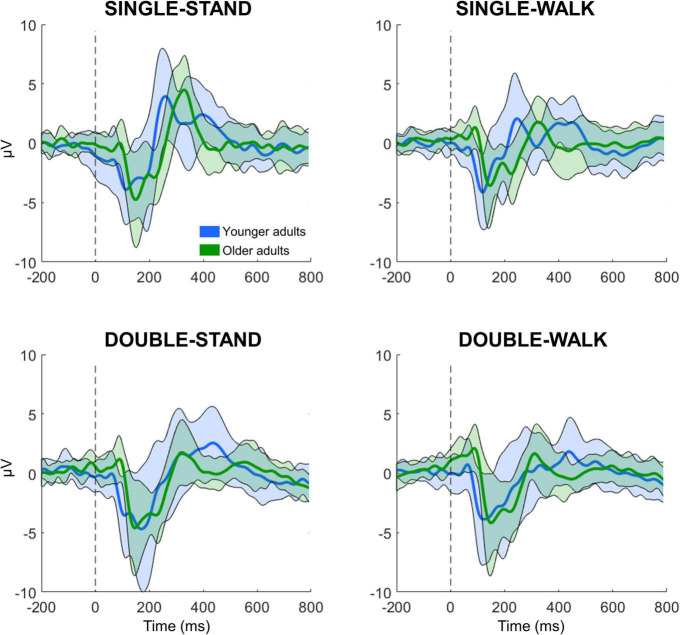
Grand averaged EEG signals from channel Pz for the younger (*n* = 21, *blue*) and older adults (*n* = 19, *green*) while performing the response time task in four different conditions: single stimulus while standing (SINGLE-STAND), single stimulus while walking (SINGLE-WALK), double stimuli while standing (DOUBLE-STAND), double stimuli while walking (DOUBLE-WALK). The thick line represents the average, and the shaded area represents ± 1 standard deviation.

All processing and analysis were performed in Matlab using an open-source toolbox for processing EEG data (EEGLAB 13.0.1b) (Delorme and Makeig, 2004). For each subject, the 16 EEG datasets (four datasets from four conditions) were merged into a single dataset, which was band-pass filtered (1–100 Hz) and line noise removal (60 Hz). EEG channels were checked for rejection using the following methods: (i) channels with magnitude < 30 or > 2,000 μV; (ii) channels with kurtosis > 5 *SD*s from the mean; and (iii) channels with standard deviation substantially greater compared to neighboring channels (Oliveira et al., 2017, 2018a). No channels were rejected from any subject in this experiment. EEG data sectors containing large amplitude fluctuations (> 5,000 μV) caused by head movement or muscle artifacts were removed from the datasets. After re-referencing the EEG data to an average reference, independent component analysis (RUNICA, from EEGLAB) was performed on the dataset to identify and remove eye blinks (Plöchl et al., 2012; Chaumon et al., 2015). The event-related potential (ERP) analysis was conducted using channels Fz, C3, Cz, C4, and Pz. These channels were chosen due to the information captured from the brain regions, which are related to executive function, motor execution (M1), sensorimotor processing (posterior parietal cortex). The merged dataset was separated into epochs for the four different experimental conditions, with an epoch length from −0.5 to 1 s window surrounding the stimulus presentation. From each epoch, the N200 and P300 peaks and latencies were extracted. The N200 is defined as the local minima from 0 to 250 ms following stimulus presentation, whereas the P300 is defined as the local maxima from 250 to 600 ms. N200 and P300 peaks and latencies are expressed in μV and ms, respectively. A total of 41 ± 4, 40 ± 3, 41 ± 2, and 41 ± 2 epochs were averaged for young adults for SINGLE-STAND, SINGLE-WALK, DOUBLE-STAND, and DOUBLE-WALK, respectively. For older adults, the number of averaged epochs was 40 ± 2, 40 ± 2, 38 ± 6, and 38 ± 3 for SINGLE-STAND, SINGLE-WALK, DOUBLE-STAND, and DOUBLE-WALK, respectively. EEG epochs from incorrect responses, as well as EEG epochs containing artifacts, were removed from the datasets. Figure 1 illustrates the grand average ERP data from all conditions for both groups at the channel Pz.

### Statistical Analysis

Data P300 amplitudes and latencies from channels C3, Cz, and C4 were averaged for each subject for representation (Malcolm et al., 2015). Statistical analysis were performed using the Statistical Package for Social Sciences (SPSS Version 24, IBM Corporation, Armonk, New York, United States) software. The normality of the dependent variables was confirmed using the Shapiro-Wilk test. Between-group differences in preferred walking speed were assessed using independent *t*-tests. The within-subject effects of stimuli (single stimuli vs. double stimuli) and posture (standing vs. walking), as well as the between-subject effects of group (younger vs. older adults) on the dependent variables (response time, N200 peak and latency, P300 peak and latency) were assessed by a 3-way repeated measure ANOVA with mixed factors. In addition, the within-subject effects of stimuli (single stimuli vs. double stimuli) and time (before vs. after stimulus presentation) as well as the between-subject effects of group (younger vs. older adults) on step frequency and the variability of step frequency and SF_*CV*_ were assessed by a 2-way repeated measure ANOVA with mixed factors. Partial eta-squared (η*_*p*_*^2^) was used to calculate the effect sizes of the statistical results, which were classified as weak (η*_*p*_*^2^ < 0.01), medium (η*_*p*_*^2^ 0.01 < 0.06), or high (η*_*p*_*^2^ > 0.14) (Cohen, 1988). Pearson correlations between reaction time and EEG variables were conducted within the same condition, to determine the relation between task performance and electrocortical activity. Correlations were classified as weak (0.2–0.39), moderate (0.4–0.59), strong (0.6–0.79), or very strong (0.8–1) (Wuensch, 1996). The significance level was set at *p* < 0.05. Data are displayed as mean ± standard deviation (SD).

## Results

### Response Time

**FIGURE 2:**
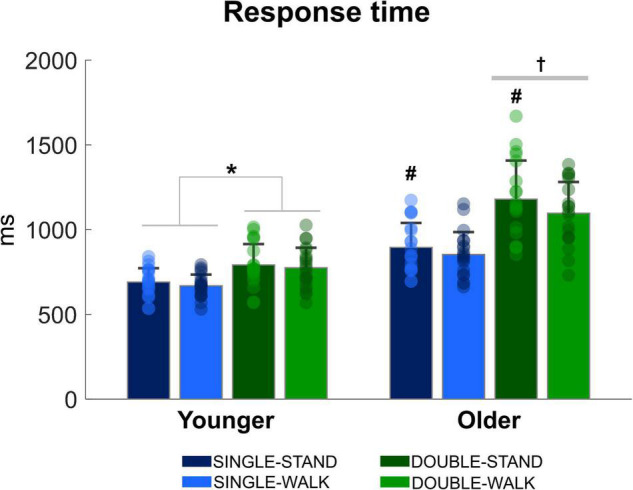
Mean (SD) and individual values of response time in four conditions: single stimulus while standing (SINGLE-STAND), single stimulus while walking (SINGLE-WALK), double stimuli while standing (DOUBLE-STAND), double stimuli while walking (DOUBLE-WALK) for younger and older adults. *Denotes significant difference between SINGLE and DOUBLE stimuli (*p* < 0.0001). ^†^Denotes significant difference in relation to single stimuli condition for older adults, and single and double stimuli conditions for young adults (*p* < 0.001). ^#^Denotes significant difference in relation to walking conditions for the same group (*p* < 0.05).

There was a stimulus x group interaction [*F*_(1, 38)_ = 38.00, *p* < 0.0001, η*_*p*_*^2^ = 0.826, Figure 2], demonstrating that double stimuli from older adults was ∼23% slower when compared to single stimuli from older adults (*p* = 0.0001), as well as compared to single stimuli (∼40%, *p* = 0.00001) and double stimuli for young adults (∼31% *p* = 0.0001). When the response time was analyzed for each group separately, it was found that the response time during double stimuli was longer when compared to single stimuli for young adults [*F*_(1, 20)_ = 58.85, *p* < 0.0001, η*_*p*_*^2^ = 0.746]. Regarding posture, older adults presented a significantly shorter response time during walking compared to standing [*F*_(1, 18)_ = 5.42, *p* < 0.05, η*_*p*_*^2^ = 0.231]. However, no effect of posture was found for young adults (*p* = 0.092). Finally, the number of correct responses in the double stimuli condition was similar between groups in standing (younger: 97.6 ± 2.3%; older: 98.2 ± 2.8%) and walking (younger: 98.2 ± 1.8%; older: 97.2 ± 3.2%). No stimulus × group interaction (*p* = 0.08) or posture × group interaction were found (*p* = 0.13).

### Walking Speed

On average, the older adults selected a preferred walking speed that was slower than that of the young participants (3.6 ± 0.5 and 4.1 ± 0.4 km/h, respectively; *p* < 0.0001).

### Step Frequency and SF_*CV*_

**FIGURE 3:**
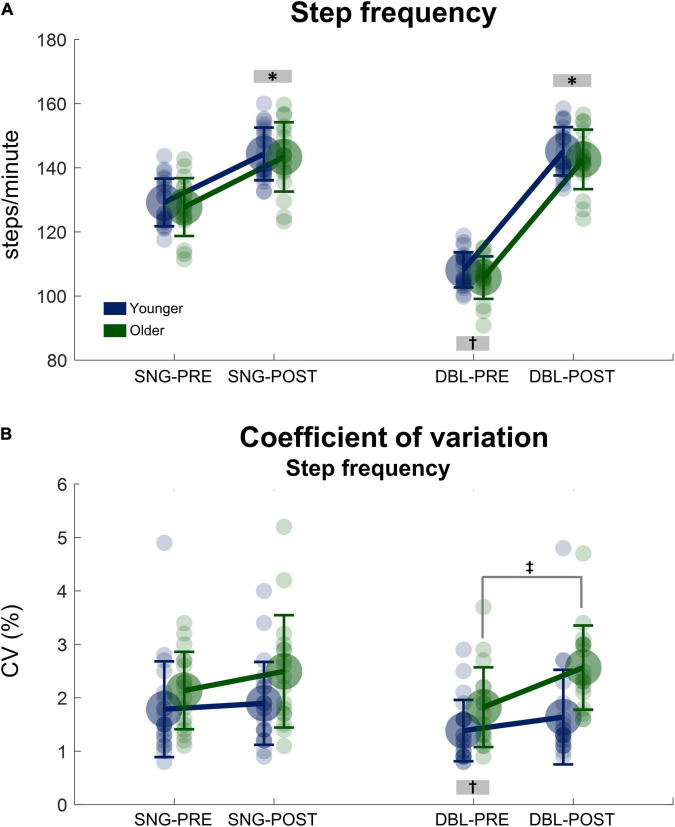
Mean (SD) and individual values of stride frequency **(A)** and the coefficient of variation of the step frequency **(B)** before (SNG-PRE) and after single stimuli (SNG-POST), as well as before (DBL-PRE) and after double stimuli (DBL-POST) for younger (*blue*) and older adults (*green*). *Denotes significant difference in relation to PRE (*p* < 0.001). ^†^Denotes significant difference in relation to DBL-POST, as well as SNG-PRE and SNG-POST (*p* < 0.0001). ^‡^Denotes significant difference from PRE to POST for the older adult group (*p* = 0.001).

There was a significant main effect of time, in which step frequency before stimulus was lower when compared to after stimulus for both groups [*F*_(1, 38)_ = 4773.29, *p* < 0.000001, η*_*p*_*^2^ = 0.15, Figure 3A]. Moreover, there was a significant stimulus x time interaction [*F*_(1, 38)_ = 5852.50, *p* < 0.000001, η*_*p*_*^2^ = 0.16] in which the step frequency before double stimuli was lower when compared to after stimuli, as well as to both PRE and POST during single stimuli (*p* < 0.000001, for all conditions). No significant effect of group was found for step frequency (*p* = 0.484).

Regarding SF_*CV*_, there was a greater SF_*CV*_ for older adults when compared to younger adults [group main effect: *F*_(1, 38)_ = 7.71, *p* < 0.01, η*_*p*_*^2^ = 0.05, Figure 3B]. However, the POST stimuli presentation results might have driven this statistical result, as *post hoc* analysis revealed no significant differences between young and older adults PRE stimuli (*p* = 0.850). A significant stimuli × time interaction was observed [*F*_(1, 38)_ = 5.94, *p* < 0.05, η*_*p*_*^2^ = 0.04], in which the SF_*CV*_ PRE double stimuli was lower when compared to POST, as well as to both PRE and POST during single stimuli (*p* < 0.001, for all conditions). Moreover, a group × time interaction was observed [*F*_(1, 38)_ = 5.182, *p* = 0.029 η*_*p*_*^2^ = 0.13, Figure 3B], but no interaction group × stimuli × time was observed [*F*_(1, 38)_ = 2,323, *p* = 0.136 η*_*p*_*^2^ = 0.62]. Therefore, a *post hoc* analysis was conducted on the single and double stimulus conditions separately. This *post hoc* analysis in the double stimuli condition demonstrated that older adults significantly increased SF_*CV*_ from PRE to POST stimuli presentation (*p* = 0.001), while no significant changes were observed in the young adults group (*p* = 0.899). Regarding single stimuli, there were no significant differences in SF_*CV*_ from PRE to POST in both age groups [interaction time × group: *F*_(1, 38)_ = 0.992, *p* = 0.326, η*_*p*_*^2^ = 0.027, Figure 3B].

### ERP—Frontal Electroencephalographic Channel

**FIGURE 4:**
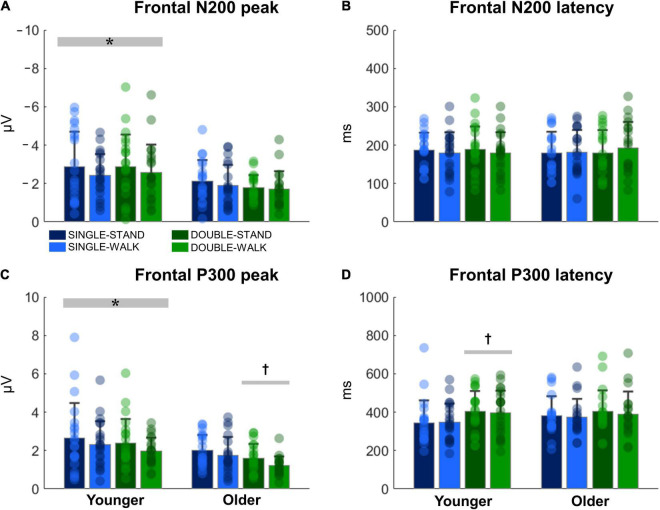
Mean (SD) and individual values of N200 peak **(A)**, N200 latency **(B)**, P300 peak **(C)**, and P300 latency **(D)** in four conditions for the channel Fz: single stimulus while standing (SINGLE-STAND), single stimulus while walking (SINGLE-WALK), double stimuli while standing (DOUBLE-STAND), double stimuli while walking (DOUBLE-WALK) for younger and older adults. *Denotes significant difference in relation to older adults (*p* < 0.0005). ^†^Denotes significant difference in relation to single stimuli conditions for the same group (*p* < 0.05).

The N200 and P300 peaks were significantly greater for young adults when compared to older adults [N200: *F*_(1, 38)_ = 14.70, *p* < 0.0005, η*_*p*_*^2^ = 0.08; P300, Figure 4A]; [*F*_(1, 38)_ = 14.08, *p* < 0.0005, η*_*p*_*^2^ = 0.08, Figure 4C]. Double stimuli induced lower P300 peaks when compared to single stimuli in older adults [*F*_(1, 18)_ = 6.51, *p* < 0.05, η*_*p*_*^2^ = 0.08, Figure 4C]. Moreover, younger adults presented longer P300 latencies when responding to double stimuli when compared to single stimuli [*F*_(1, 20)_ = 4.74, *p* < 0.05, η*_*p*_*^2^ = 0.05, Figure 4D]. However, no changes in N200 latencies were found (Figure 4B). There were no effects of posture for any variable for channel Fz (N200 peak: *p* = 0.28; N200 latency: *p* = 0.52; P300 peak: *p* = 0.24; P300 latency: *p* = 0.91). Moreover, no stimulus × posture interaction was found (N200 peak: *p* = 0.80; N200 latency: *p* = 0.92; P300 peak: *p* = 0.83; P300 latency: *p* = 0.86).

### ERP—Central Channels

**FIGURE 5:**
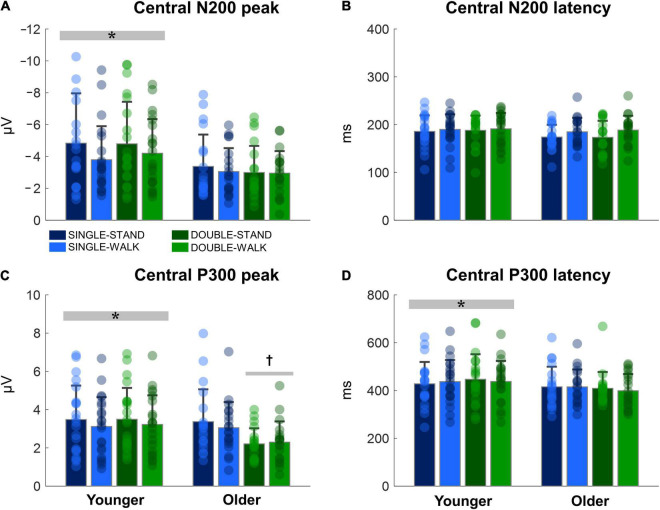
Mean (SD) and individual values of N200 peak **(A)**, N200 latency **(B)**, P300 peak **(C)**, and P300 latency **(D)** in four conditions for the averaged central channels (C3, Cz, and C4): single stimulus while standing (SINGLE-STAND), single stimulus while walking (SINGLE-WALK), double stimuli while standing (DOUBLE-STAND), double stimuli while walking (DOUBLE-WALK) for younger and older adults. *Denotes significant difference in relation to older adults (*p* < 0.05). ^†^Denotes significant difference in relation to single stimuli conditions for the same group (*p* < 0.005).

The N200 and P300 peaks were significantly greater for young adults when compared to older adults [N200: *F*_(1, 38)_ = 14.18, *p* < 0.0005, η*_*p*_*^2^ = 0.08; P300, Figure 5A]; [*F*_(1, 38)_ = 6.13, *p* < 0.05, η*_*p*_*^2^ = 0.03, Figure 5C]. No changes in N200 latencies were found (Figure 5B). The P300 latency was significantly longer for young adults when compared to older adults [*F*_(1, 38)_ = 4.16, *p* < 0.05, η*_*p*_*^2^ = 0.025; P300, Figure 5D]. Moreover, double stimuli induced lower P300 peaks when compared to single stimuli in older adults [*F*_(1, 18)_ = 10.13, *p* < 0.005, η*_*p*_*^2^ = 0.12, Figure 5C]. There were no effects of posture for any variable for the central channels (N200 peak: *p* = 0.15; N200 latency: *p* = 0.60; P300 peak: *p* = 0.38; P300 latency: *p* = 0.99). Moreover, no stimulus × posture interaction was found (N200 peak: *p* = 0.69; N200 latency: *p* = 0.94; P300 peak: *p* = 0.89; P300 latency: *p* = 0.66).

### ERP—Parietal Channel

**FIGURE 6:**
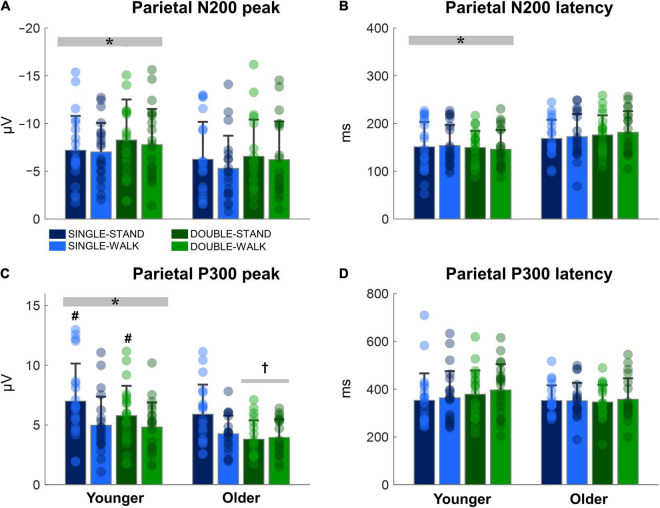
Mean (SD) and individual values of N200 peak **(A)**, N200 latency **(B)**, P300 peak **(C)**, and P300 latency **(D)** in four conditions for the channel Pz: single stimulus while standing (SINGLE-STAND), single stimulus while walking (SINGLE-WALK), double stimuli while standing (DOUBLE-STAND), double stimuli while walking (DOUBLE-WALK) for younger and older adults. *Denotes significant difference in relation to older adults (*p* < 0.05). ^†^Denotes significant difference in relation to single stimuli conditions for the same group (*p* < 0.05). ^#^Denotes significant difference in relation to walking conditions for the same group (*p* < 0.05).

The N200 and P300 peaks were significantly greater for young adults when compared to older adults [N200: *F*_(1, 38)_ = 6.14, *p* < 0.05, η*_*p*_*^2^ = 0.03; P300, Figure 6A]; P300: [*F*_(1, 38)_ = 9.32, *p* < 0.005, η*_*p*_*^2^ = 0.05, Figure 6C]. In addition, the N200 latency was significantly shorter for young adults when compared to older adults [*F*_(1, 38)_ = 12.66, *p* < 0.0005, η*_*p*_*^2^ = 0.07, Figure 6B]. Double stimuli induced lower P300 peaks when compared to single stimuli in older adults [*F*_(1, 18)_ = 7.81, *p* < 0.01, η*_*p*_*^2^ = 0.08, Figure 6C]. Finally, the P300 peak was smaller during the walking tasks when compared to standing for young adults [*F*_(1, 17)_ = 4.74, *p* < 0.05, η*_*p*_*^2^ = 0.05, Figure 6C]. No effects of posture were found for older adults (N200 peak: *p* = 0.46; N200 latency: *p* = 0.64; P300 peak: *p* = 0.08; P300 latency: *p* = 0.77, Figure 6D). Moreover, no stimulus x posture interaction was found (N200 peak: *p* = 0.73; N200 latency: *p* = 0.94; P300 peak: *p* = 0.19; P300 latency: *p* = 0.72).

### Association Between Reaction Time and Electroencephalographic Measures

There were several moderate and significant correlations between reaction time and EEG measures for young adults (Table 1). The reaction time from SINGLE-STAND and SINGLE-WALK were correlated with Fz N200 latency from the same condition (SINGLE-STAND: *r* = 0.58, SINGLE-WALK: *r* = 0.45). Moreover, the reaction time from DOUBLE-STAND was correlated with the P300 peak from Cz (*r* = 0.52) and Pz (*r* = 0.63), as well as P300 latency from Pz (*r* = −0.47). Finally, the reaction time from DOUBLE-WALK was correlated with the P300 latency from Pz (*r* = 0.48). Regarding older adults, moderate and significant correlations were found only between DOUBLE-STAND reaction time and P300 latency in Cz from the same condition (*r* = −0.52), and between SINGLE-WALK reaction time and P300 peak from Pz (*r* = −0.46). No significant correlations were found between reaction time from SINGLE-STAND or DOUBLE-WALK with any EEG variables for older adults. The correlations not included in the table were computed, but no significant association has been found.

**TABLE 1:** Pearson correlation coefficients (r) and significance values (p) from the correlations between reaction time and EEG variables during single stimulus while standing (SINGLE-STAND), single stimulus while walking (SINGLE-WALK), double stimuli while standing (DOUBLE-STAND), double stimuli while walking (DOUBLE-WALK) for younger and older adults.

	Condition	EEG variable	*r*	*p*
**Young adults**	SINGLE-STAND	Fz N200 latency	0.582	0.006
	SINGLE-STAND	Pz P300 peak	0.552	0.010
	DOUBLE-STAND	C P300 peak	0.525	0.015
	DOUBLE-STAND	Pz P300 peak	0.632	0.002
	DOUBLE-STAND	Pz P300 latency	–0.475	0.030
	SINGLE-WALK	Fz N200 latency	0.455	0.038
	SINGLE-WALK	Pz P300 latency	–0.473	0.031
	DOUBLE-WALK	Pz P300 latency	–0.487	0.025
**Older adults**	DOUBLE-STAND	C P300 latency	0.521	0.022
	SINGLE-WALK	Pz P300 peak	–0.460	0.048

## Discussion

Our main findings revealed that responding to a double stimuli (e.g., decision making) during walking increased response time and step frequency variability following stimulus presentation, particularly in older adults. Interestingly, the P300 amplitudes of older adults were largely influenced by the complexity of the cognitive task (single × double stimuli) rather than by posture (standing × walking) in regions related to cognitive function, motor performance, and somatosensory integration. Moreover, the relation between response time and electrocortical activity in the frontal and parietal regions was present only in young adults, whereas the limited number of significant correlations in older adults exemplifies the need for relocation of brain processes to complete different tasks. These findings suggest that physically active older adults present greater impairments in walking performance when the dual-task involves decision making. Moreover, the demand for brain resources to accomplish the decision-making while walking influences electrocortical activity across different brain regions.

### Response Time During Walking

Overall, physically active older adults were slower to respond to stimuli when compared to young adults, which may be explained by age-related changes in neuromuscular function (Pollock et al., 2015), cognitive control, and efficiency of information processing (Baltes and Lindenberger, 1997; Hedden and Gabrieli, 2004). Concerning response time during walking, several studies have demonstrated impairments in gait performance when the complexity of cognitive tasks increase in dual-task during walking either in young (Lövdén et al., 2008; Beurskens et al., 2014; Malcolm et al., 2015) or in older adults (Lövdén et al., 2008; Beurskens et al., 2014; Oliveira et al., 2018b). While the effect of cognitive load on walking has been extensively investigated, the reciprocal effect of walking on cognitive performance received much less attention (Schaefer et al., 2010). In the present study, the walking task did not negatively influence the response time performance, either during the single or double stimuli. In fact, physically active older adults improved their response times when both cognitive conditions were performed while walking when compared to standing, as similarly described previously by this research group for young adults (Oliveira et al., 2018b). Despite an expected reduction in motor performance under complex cognitive conditions, the performance of sensorimotor tasks can increase arousal levels and improve the performance of a concurrent cognitive task (McMorris and Graydon, 2000; Schaefer et al., 2010). Our results indicated that physically active older adults were able to adapt their motor system to respond to the double stimuli while walking without loss of balance or slowed response time. Moreover, the maintenance of performance in the response time task may be related to improvements in arousal levels evoked during walking (Lövdén et al., 2008; Schaefer et al., 2010) for both groups, but especially for older adults. Our results demonstrated that decision-making tasks, rather than walking tasks, induced greater cognitive costs in physically active older adults. Interestingly, the motor action of walking seems to assist in the maintenance of response time performance by increasing arousal levels. However, task complexity can affect walking performance as demonstrated by our results on step variability.

### Age- and Task Complexity-Related Effects on Step Frequency

Both young and older adults reduced their step frequency prior to the presentation of the stimulus that required decision-making (double-stimuli), suggesting that participants adopted a walking strategy based on longer steps. Our results corroborate those from previous studies reporting increased stride length while performing complex dual-task during walking in young (De Sanctis et al., 2014; Malcolm et al., 2015; Oliveira et al., 2018b) and older adults (Lövdén et al., 2008; Li et al., 2012). A reduced number of steps per unit of time can reduce inter-task competition by increasing desynchronization between tasks, freeing cognitive resources to perform a more complex cognitive task (De Sanctis et al., 2014; Malcolm et al., 2015). It has been reported that the sharing of attentional resources in a dual-task walking paradigm may lead to decreased speed, decreased stride length, and increased stride time variability (Faulkner et al., 1993; Lövdén et al., 2008; Beurskens et al., 2014; Oliveira et al., 2018b). Conversely, it has been previously shown that motor performance during walking can be improved in the presence of a concurrent cognitive task (Lövdén et al., 2008; Schaefer et al., 2010). A decline in stride time variability was observed either in young (Lövdén et al., 2008; Schaefer et al., 2010) and older adults (Lövdén et al., 2008), while cognitive performance remained unaltered (Lövdén et al., 2008; Schaefer et al., 2010). The presentation of visual stimuli during walking may be considered a perturbation to dynamic balance, and our results revealed that older adults increased step frequency variability following stimulus presentation, especially in the double-stimuli condition. Therefore, more complex cognitive stimuli marginally influences the walking patterns of physically active young adults, but it may disrupt walking patterns even in physically active older adults. However, it is noteworthy that our walking task was performed while predominantly having the hands resting on a table. This action may have improved the gait stability of our participants, as it has been shown that light tactile feedback can improve postural stability (Jeka, 1997; Albertsen et al., 2010). Thus, comparing our results to those from stereotyped walking recordings should be done with caution.

### Age- and Task Complexity Effects on Electrocortical Activity

Through the use of surface EEG, it is possible to investigate changes in early (N200) and later stages (P300) of cortical responses to stimuli (Polich, 2007; Huang et al., 2015). N200 events are termed “sensory,” as they depend primarily on the physical parameters of the stimulus (Huang et al., 2015). In contrast, P300 events are larger in amplitude and are termed “cognitive,” as they examine sensory information processing by the brain (Polich, 2007; Huang et al., 2015). Our results demonstrated that both N200 and P300 peaks were reduced in older adults when compared to younger adults in all brain regions, corroborating previous reports on a gradual reduction in P300 amplitudes from early adulthood to old age (Polich, 2007). Also, changes in the P300 latency have been observed across the lifespan (Polich, 2007). Moreover, P300 is sensitive to task processing demands and varies with individual differences in cognitive capability (Huang et al., 2015). Several aspects might account for a decline in electrocortical activity in older adults. Anatomically, it has been shown that the association cortices, the neostriatum, and the cerebellum are profoundly affected by aging (Hedden and Gabrieli, 2004; Kennedy and Raz, 2009). Moreover, lesser volumes of the prefrontal cortex and the hippocampus regions have been associated with altered brain activation (Daselaar et al., 2015) and poorer performance on executive function tasks (Reuter-Lorenz and Lustig, 2005; Kennedy and Raz, 2009).

Regarding task complexity, the double-stimuli response time task induced a decline of the P300 amplitudes across all brain regions in older adults, regardless of the motor task involved. On the other hand, young adults did not change their peak amplitudes significantly, but their frontal P300 latency was longer when performing the decision-making task. Our results corroborate the suggestion that P300 events are related to cognitive processing and can help underpinning the cognitive costs related to dual-tasks during walking (Polich, 2007; Huang et al., 2015). Despite the increasing number of studies evaluating ERPs during walking, there are no direct studies to compare with our experimental design, which involved upper limb response time performance at simple and complex (e.g., decision-making) levels during walking. Previous studies evaluating dual-task during walking classify a simple cognitive task as a task performed while standing, whereas a complex task combines the cognitive task with walking (Malcolm et al., 2015; Maidan et al., 2019; Reiser et al., 2019). The age-related changes in dual-task performance between stationary (standing or sitting) and walking performance have been proposed in go/No-go tasks (Malcolm et al., 2015) and oddball tasks (Maidan et al., 2019; Reiser et al., 2019). Malcolm et al. (2015) revealed that young adults present reduced P300 amplitude during a Go/No-Go task during walking, while older adults presented a slight increase. Moreover, young adults reduced P300 latencies during the dual-task walking compared to single walking, while no changes in P300 latencies were observed for older adults (Malcolm et al., 2015).

In another study, Maidan et al. (2019) revealed that P300 latencies at Pz were significantly longer for both young and older adults during an oddball task performed while walking, and older adults presented longer P300 latencies than young adults. On the other hand, the dual-task paradigm did not alter P300 amplitude within each age group, and no differences between young and older adults were observed. Our findings are not aligned with such results, as older adults presented reduced P300 amplitudes without changes in P300 latencies during the decision-making task. Conversely, younger adults presented no changes in P300 amplitudes but P300 latencies increased at the frontal region. Therefore, our results suggest that walking influences ERP modulation only in young adults, and regardless of the cognitive task complexity. This finding is in line with Reiser et al. (2019), who found that the P300 amplitude from channel Pz in young adults is reduced during walking when compared to standing during an auditory oddball task. Similar results were observed by Ladouce et al. (2019), who demonstrated reductions in parietal (Pz) P300 amplitude during an auditory oddball task during walking when compared to a standing position. Furthermore, the impact is significantly greater when participants walked on a hallway than on a treadmill. Interestingly, this study showed that walking on a hallway or being wheeled through the same hallway produced similar attenuations in P300 amplitudes. The implemented experimental design allowed the authors to conclude that the decline in P300 amplitudes was not caused by the act of walking *per se* but rather by the linear and additive sum of the processing demands produced by visual and inertial stimulation (Ladouce et al., 2019). These results suggest that the overall effect on attention is likely to reflect the linear sum of all stimulation sources independently of the task context and goals.

Our results regarding N200 were very similar to those from P300: young adults presented higher amplitudes than older adults, and the complexity of the cognitive task did not alter either P200 amplitude or latency. In addition, posture (standing vs. walking) did not influence N200. This lack of modulation on N200 under different cognitive complexity and postures partially contradicts Malcolm et al. (2015), as their study showed a robust N200 amplitude reduction while walking, particularly over central-parietal sites for young adults. On the other hand, Malcolm et al. (2015) did not report changes in N200 in older adults, which corroborates our findings across the different postures and cognitive complexity tasks. Interestingly, young adults presented significant correlations between the Fz N200 latency and reaction time in the single stimuli task during both standing and walking, indicating that this early brain process related to sensory control may be strictly related to accomplishing simple cognitive reaction time. However, older adults did not present any correlation with Fz, indicating that centralization of brain resources might not be possible with aging, redistributing the cognitive processing to other brain areas.

There are some explanations for the lack of agreement between our results and the presented literature in EEG experiments. It is plausible that the activation of neural networks associated with different cognitive tasks might produce different ERP modulations, causing substantial differences in divergent experimental protocols. In addition, our study presented two layers of complexity while performing a walking task (single stimuli vs. double stimuli), which may help explaining the lack of similar results with respect to previous literature. More importantly, all participants in our study were physically active, whereas the cited studies do not report the physical status of their samples. Regular physical exercise has been recommended to preserve physical and cognitive performance (Schäfer et al., 2006; Barnes, 2015; Godde and Voelcker-Rehage, 2017). In addition, older adults with higher fitness levels present shorter ERP latencies, stronger central inhibition, and better neurocognitive performance (Dustman et al., 1990; McDowell et al., 2003), as well as reduced need for cognitive resources to accomplish motor tasks (Godde and Voelcker-Rehage, 2017). Moreover, it has been shown that physically active older adults may perform similarly to young adults in simple cognitive tasks (Oliveira et al., 2018b). The greatest difference between younger and older adults in our study was related to the decision-making, regardless of the postural demands (standing or walking), as described previously (Oliveira et al., 2018b). Our results contribute to the field by demonstrating that physically active older adults present reduced N200 and P300 amplitudes across different brain regions when facing a decision-making task either while standing or walking. Older adults presented less ERP modulation, and there were some significant associations between reaction times and P300 peaks (see section “Results”). Behavioral results are usually related to changes in resource allocation, but in our study it is plausible that the reduced ERP modulation, associated with a reduced motor performance might represent a loss in flexible resource allocation across multiple tasks (Malcolm et al., 2015). Such brain activity patterns might contribute to increased motor-cognitive costs related to age. In our study older adults presented a ∼2-fold larger influence of decision making on their response times (+ 23% vs. 12% increases for older and younger people, respectively). This evidence underpins the relevance of cognitive training to maintain cognitive performance, as regular physical activities may not maintain and/or improve such neural features.

Our study presents some limitations. Firstly, we compared the cognitive-motor performance between physically active older and younger adults. Therefore, our conclusions regarding age-related differences in our study are limited to physically active older adults. This study would have benefited by the inclusion of a group of sedentary older adults, which would provide a robust baseline toward understanding the effect of physical activity on the cognitive-motor interference in older adults. Secondly, the walking task has been performed on a treadmill, which is known to influence postural control (Tong et al., 2020). Performing the task using overground walking would be preferable, but the nature of our response time task required a motor response by touching a monitor. Future studies implementing auditory tasks in a natural environment, as explored by Reiser et al. (2019), may be relevant to increase the ecological validity of this protocol. Another limitation is that the upper limb motor task might introduce movement artifacts into the EEG recordings. Despite the fact that participants were asked to avoid moving their heads when performing the reaching task, the presence of artifacts related to the arm movement cannot be discarded. Nonetheless, the data processing steps taken to minimize the effects of movement artifacts on our results were effective. Finally, the current study was limited to the use of only five EEG channels, substantially reducing the possibility of applying sophisticated methods to extract information from scalp EEG recordings. The use of independent component analysis has been highly relevant for unraveling neural information in mobile recording conditions when high-density EEG (>32) electrodes are used (Oliveira et al., 2016, 2017). In this study, independent component analysis has been used for data pre-processing and cleaning (Chaumon et al., 2015). Therefore, further studies applying high-density EEG on training protocols related to upper limb motor tasks can substantially advance our knowledge on electrocortical signatures of motor control and learning.

In summary, our study demonstrated that decision-making during a walking task slows response time and increases step frequency variability after the stimulus in physically active older adults. Moreover, older adults present an overall reduction in both N200 and P300 amplitudes in areas related to cognitive function, motor performance, and somatosensory integration when compared to young adults. More importantly, the P300 amplitudes and latencies of older adults were largely influenced by the complexity of the cognitive task (single vs. double cognitive stimuli) rather than by motor task (upright standing vs. walking at preferred pace). These deficits in cortical control for older adults might be related to relocation of cognitive processing to different brain regions, which were exemplified by the highly limited amount of correlations between reaction time and EEG variables. Therefore, physical activities may contribute to the maintenance of cortical control of cognitive performance when performing simple dual-task while walking in older adults, but electrocortical activity and subsequent cognitive-motor performance in dual-task walk involving decision-making is compromised.

## Data Availability Statement

The raw data supporting the conclusions of this article will be made available by the authors, without undue reservation.

## Ethics Statement

The studies involving human participants were reviewed and approved by the Polytechnic of Guarda Committee on Research Ethics. The patients/participants provided their written informed consent to participate in this study.

## Author Contributions

CV-C was involved in the conceptualization of the study, collecting, EEG analysis, and interpretation of the data and drafting, and revising the manuscript for intellectual content. CV was involved in the conceptualization of the study, collecting data, and revising the manuscript for intellectual content. AO was involved in the conceptualization of the study, data collection, EEG data analysis, data interpretation, and revising the manuscript for intellectual content. All authors contributed to the article and approved the submitted version.

## Conflict of Interest

The authors declare that the research was conducted in the absence of any commercial or financial relationships that could be construed as a potential conflict of interest.

## Publisher’s Note

All claims expressed in this article are solely those of the authors and do not necessarily represent those of their affiliated organizations, or those of the publisher, the editors and the reviewers. Any product that may be evaluated in this article, or claim that may be made by its manufacturer, is not guaranteed or endorsed by the publisher.
